# Unusual clinical spectra of childhood severe malaria during malaria epidemic in eastern Uganda: a prospective study

**DOI:** 10.1186/s12936-023-04586-3

**Published:** 2023-06-01

**Authors:** Cate Namayanja, Egiru Emma Isaiah Eregu, Paul Ongodia, Charles Benard Okalebo, William Okiror, Francis Okello, Ambrose Okibure, George Paasi, Hellen Kakungulu, Abongo Grace, Rita Muhindo, Duncan Banks, Chebet Martin, Simon Taylor-Robinson, Peter Olupot-Olupot

**Affiliations:** 1 Mbale Clinical Research Institute, P.O. Box 1966, Mbale, Uganda; 2 Department of Pediatrics and Child Health, Busitema University Faculty of Health Sciences, Mbale, Uganda; 3 Department of Community and Public Health, Busitema University Faculty of Health Sciences, Mbale, Uganda; 4 Varimetrics Group Limited, Mbale, Uganda; 5 Imperial College London, London, UK; 6 Busitema University, TORORO, Uganda; 7 The Open University, Milton Keynes, UK

**Keywords:** Clinical spectrum, Severe malaria, Child, Prolonged hospitalization and mortality

## Abstract

**Background:**

In sub-Saharan Africa (SSA), malaria remains a public health problem despite recent reports of declining incidence. Severe malaria is a multiorgan disease with wide-ranging clinical spectra and outcomes that have been reported to vary by age, geographical location, transmission intensity over time. There are reports of recent malaria epidemics or resurgences, but few data, if any, focus on the clinical spectrum of severe malaria during epidemics. This describes the clinical spectrum and outcomes of childhood severe malaria during the disease epidemic in Eastern Uganda.

**Methods:**

This prospective cohort study from October 1, 2021, to September 7, 2022, was nested within the ‘Malaria Epidemiological, Pathophysiological and Intervention studies in Highly Endemic Eastern Uganda’ (TMA2016SF-1514-MEPIE Study) at Mbale Regional Referral Hospital, Uganda. Children aged 60 days to 12 years who at admission tested positive for malaria and fulfilled the clinical WHO criteria for surveillance of severe malaria were enrolled on the study. Follow-up was performed until day 28. Data were collected using a customized proforma on social demographic characteristics, clinical presentation, treatment, and outcomes. Laboratory analyses included complete blood counts, malaria RDT (SD BIOLINE Malaria Ag P.f/Pan, Ref. 05FK60-40-1) and blood slide, lactate, glucose, blood gases and electrolytes. In addition, urinalysis using dipsticks (Multistix^®^ 10 SG, SIEMENS, Ref.2300) at the bedside was done. Data were analysed using STATA V15.0. The study had prior ethical approval.

**Results:**

A total of 300 participants were recruited. The median age was 4.6 years, mean of 57.2 months and IQR of 44.5 months. Many children, 164/300 (54.7%) were under 5 years, and 171/300 (57.0%) were males. The common clinical features were prostration 236/300 (78.7%), jaundice in 205/300 (68.3%), severe malarial anaemia in 158/300 (52.7%), black water fever 158/300 (52.7%) and multiple convulsions 51/300 (17.0%), impaired consciousness 50/300(16.0%), acidosis 41/300(13.7%), respiratory distress 26/300(6.7%) and coma in 18/300(6.0%). Prolonged hospitalization was found in 56/251 (22.3%) and was associated with acidosis, *P* = 0.041. The overall mortality was 19/300 (6.3%). Day 28 follow-up was achieved in 247/300 (82.3%).

**Conclusion:**

During the malaria epidemic in Eastern Uganda, severe malaria affected much older children and the spectrum had more of prostration, jaundice severe malarial anaemia, black water fever and multiple convulsions with less of earlier reported respiratory distress and cerebral malaria.

## Background

Malaria is an acute febrile disease caused by any human-infecting *Plasmodium* species; in sub-Saharan Africa (SSA), *Plasmodium falciparum* is responsible for most of the malaria morbidity, including severe disease, which is often fatal. This species is also responsible for epidemics, but the clinical spectrum of severe malaria during epidemics is poorly described. The 2020 World Malaria Report of the World Health Organization (WHO) reported an increase in the estimated number of malaria cases from 227 million in 2019 to 241 million in 2020 in 85 malaria-endemic countries [1]. In 2022, the estimated malaria cases were 247 million. Of the 85 malaria endemic countries globally, 26(30.6%) disproportionately contributed to 96% of the global disease case burden that year. Moreover, the WHO Afro Region with six malaria high-burden countries, including Nigeria (26.8%), the Democratic Republic of Congo (12%), Uganda (5.4%), Mozambique (4.2%), Angola (3.4%), and Burkina Faso (3.4%) contributed to approximately 55% of all the mortality due to the disease in 2020. The COVID-19 pandemic is thought to have disrupted the healthcare delivery system, especially in the WHO Afro Region, where more than 69,000 malaria excess deaths were registered [2].

**Fig. 1:**
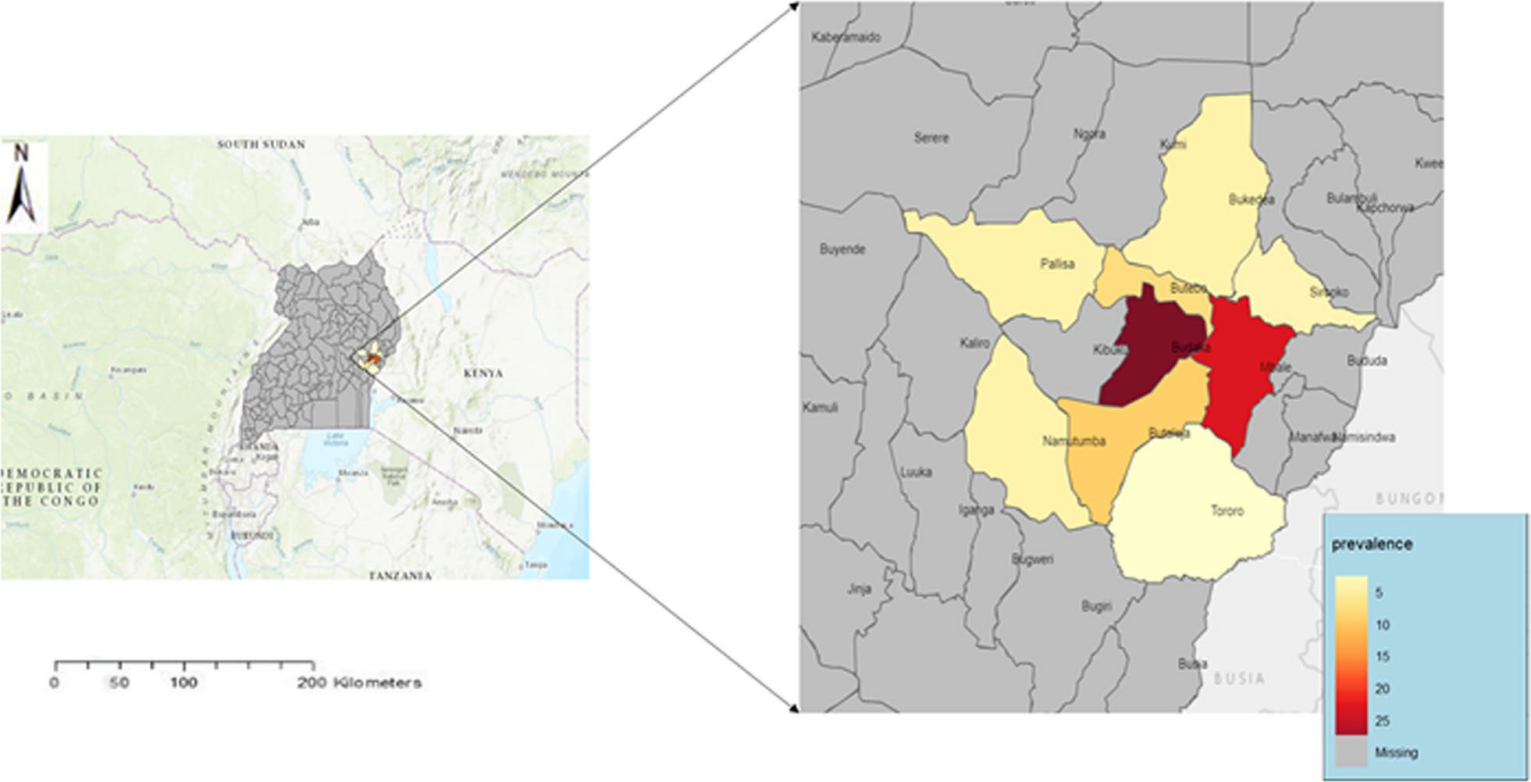
Map of eastern Uganda showing districts experiencing malaria epidemic

Uganda has 90% of its population at risk of malaria [3]. On average, the national prevalence of malaria is 9%, but varying from region to region with the lowest in Kampala, in the central region at 0.3% and the highest in Karamoja in the northeastern region at 34% [4]*.* Eastern Uganda is known to have perennial transmission where estimated entomological inoculation rates (EIR) are greater than 100 bites per person per year [5]. Previous studies have found that in Eastern Uganda, severe malaria commonly presents with respiratory distress, hyperparasitaemia, jaundice, severe anaemia, hyperlactatemia and dark urine [5]. However, these studies were done more than 10 years ago; it is unclear whether the clinical spectrum of severe malaria in Uganda has changed. In June 2022, the region experienced a malaria epidemic [6]. During this epidemic, there were unprecedented numbers of children admitted with manifestations of black water fever (BWF), a life-threatening condition, which clinicians have noted is associated with acute kidney injury but has not been systematically studied. Figure 1 below shows the districts that experienced the epidemic in Eastern Uganda.

This study described the clinical spectrum and outcomes of severe malaria among children admitted to Mbale Regional Referral Hospital (Mbale RRH) in Eastern Uganda. The working hypothesis for this study was; the epidemiological transition of malaria has changed the clinical spectrum of severe malaria outcomes.

## Methods

The study was a prospective study as part of the ‘Malaria Epidemiological, Pathophysiological and Intervention studies in Highly Endemic Eastern Uganda’ (TMA 2016SF-1514-MEPIE Study) at Mbale RRH. This study targeted patients aged 2 months to 12 years, admitted with features of severe *P. falciparum* malaria. The disease was surveilled using the WHO clinical criteria [7], previously used for the same purposes in the study area [5]. These included severe anaemia (Hb less than 5 g/dL), prostration (generalized weakness so that the person is unable to sit, stand or walk without assistance), shock (compensated shock was capillary refill time ≥ 3 s or temperature gradient and no hypotension). Decompensated shock is defined as systolic blood pressure < 70 mmHg in children with evidence of impaired perfusion (cool peripheries or prolonged capillary refill), spontaneous haemorrhage (recurrent or prolonged bleeding from nose, gums or venipuncture sites; haematemesis or melaena), multiple convulsions (2 or more convulsions in 24 h), impaired consciousness (Blantyre coma score < 3), jaundice (plasma bilirubin > 50umol/L (> 3 mg/dL), pulmonary oedema/respiratory distress (Kussmaul’s breathing manifesting as deep breathing with signs of increased work of breathing) and (haemoglobinuria), hyperparasitaemia (greater than 10% in highly endemic areas), acidosis (venous plasma lactate > 5 mmol/L), hypoglycaemia (< 2.2 mmol/L or < 40 mg/dL), lactic acidosis (lactate ≥ 5 mol/L), renal failure (serum creatinine > 3 mg/dL/BUN > 20 mmol/L) [5].

Sample size of 300 was derived using the Kish formula. Study period was from October 1, 2021, to September 7, 2022.

### Patient eligibility

At the TMA 2016SF-1514-MEPIE Study, all children presenting for admission at the Acute Care Unit (ACU) of Mbale RRH were screened for malaria using a malaria rapid diagnostic test (RDT). Children with positive RDT and any one feature of severe/complicated malaria [5] were tested for malaria using a 10% Giemsa-stained blood slide to confirm the diagnosis of asexual *P. falciparum* parasitaemia. Those with a positive blood slide for *P. falciparum* were randomly selected among children being recruited into a longitudinal malaria study (MEPIE). Study clinicians (Nurses and Medical Officers) consented participants in the study through a two-stage process as per protocol.

### Study procedures

The study procedures were conducted in the ACU ward of Mbale RRH and the laboratory procedures at Mbale Clinical Research Institute [MCRI (www.mcri.ac.ug)]. A history of the illness was taken, and qualified medical officers also trained on the study protocol did a clinical examination. Blood and urine samples were then taken for bedside as well as laboratory tests. In the ward, some of the bedside procedures included screening for malaria using RDT SD—Bioline^™^ malaria—Ag-pf/pan (Standard Diagnostics Inc, Korea, Ref. 05KF60), random blood sugar assays (RBS) (mmol/L) (Nova Biomedical Corporation, Waltham, MA, USA), lactate (mmol/L) (Nova Biomedical Corporation, Waltham, MA, USA) and urine dipsticks (Multistix^®^ 10 SG, SIEMENS, Ref. 2300). In addition, i-STAT CHEM8 + (Abbott Diagnostics, Stockport, UK) was used to measure blood sodium, potassium, chloride, total carbon dioxide, anion gap, ionized calcium, glucose, urea nitrogen, creatinine, lactate, haematocrit and haemoglobin. Qualified and certified laboratory staff carried out blood test analyses using a Beckman Coulter DxH-500 haematology analyser (Beckman Coulter Ltd, High Wycombe, UK), while biochemistry was performed using a Cobas c111 analyser (Roche Diagnostics PTY, Midrand, South Africa). Blood slides were examined at MCRI laboratory with quality control undertaken by experienced microscopists. Haemoglobin was obtained using HemoCue^™^ Hb 301 (Hemocue AB, Angelholm Sweden). Lastly, urine dipstick results were obtained using Siemens multistix10 SG reagent strips (Siemens Healthcare Diagnostics Inc, Tarrytown, USA).

### Quality control, data management and analysis

Research assistants were trained in data collection methods. All data from this study were cleaned and analysed using STATA^™^ version 15 (STATA Corporation, College Station, TX, USA). The database was designed according to the outline and sections of the questionnaire for easy data entry and resolution of data queries. Data were captured and entered using Redcap V12.2.1 (Vanderbilt University, Tennessee, USA) by GCP-trained data assistants. Data were presented using frequency tables and proportions. Data analysis was in three stages, the univariate included frequencies, percentages and continuous variables reported as measures of central tendency as mean, median and interquartile range (IQR) depending on the distribution. Bivariate analysis we reported cross tabulations between the dependent variable (mortality and prolonged hospital stay) against the independent variables (the severe malaria clinical spectrum) and reported the chi-square. At multivariate table, a logistic regression reporting odds ratios and respective p-values.The study had ethical approval by Mbale Regional Referral Hospital Research Ethics committee (MRRH-REC) REC #MRRH-2021-76 prior to the data collection.

## Results

A total of 300 eligible participants aged 60 days to 12 years were enrolled from October 1, 2021, to August 2, 2022.

### Social demographic characteristics

The median age in this study was 4.6 years, mean of 57.2 months and IQR of 44.5 months. In total, 164/300 (54.7%) were under 5 years. More than half, 171/300 (57.0%), were males. Many of the participants were from areas where the malaria epidemic was reported by the Uganda Ministry of Health, including the districts of; Budaka 88/300 (29.3%), Mbale 68/300 (22.7%), Butaleja 28/300 (9.3%), Butebo 23/300 (7.7%), Kibuku 26/300 (8.7%), Namutumba 12/300 (4.0%), Bukedea 12/300 (4.0%), Sironko 10/300 (3.3%), Tororo 4/300 (1.3%) and Pallisa (12/300 (4.0%); in Eastern Uganda.

### Severe malaria spectrum

Fever was present in 295/300 (98.3%). Prostration was the most commonly observed presentation in 236/300 (78.7%), of whom 134/236 (55.0%) had severe anaemia, and 76/134 (56.7%) received a blood transfusion immediately at admission.

Jaundice was the second commonest clinical feature and was observed in 205/300 (68.3%) participants. Of these, 124/205 (60.5%) had severe malaria anaemia, 144/205 (70.2%) had reported abdominal pain and 152/205 (74.1%) had reported vomiting. The majority of those with jaundice, 42/205 (20.5%), had an elevated LDH, 95/197(48.2%) leukocytosis, and 50/197 (25.3%) had thrombocytopenia.

Severe malaria anaemia was observed in 158/300 (52.7%) participants, while 104/158 (65.8%) had reported abdominal pain, 107/158 (67.7%) had vomiting, and 62/158 (39.2%) were reported to have passed dark urine. Tachycardia was present in 64/75 (85.3%), 17/158 (10.8%) had respiratory distress, and 16/158 (10.1%) had a weak pulse.

Haemoglobinuria was observed in 158/300 (52.7%) participants, the majority of whom were males 99/158(62.7%), and those 5 years or more were 95/158 (60.1%). A sizeable number—116/158 (73.4%) had reported abdominal pain, and 126/158 (79.7%) reported vomiting. Over half 118/158 (74.7%), had two or more admissions in the previous year. In this group with haemoglobinuria, 139/158 (88.0%) had jaundice and 86/158 (54.4%) had severe anaemia.

Multiple convulsions were observed in 51/300 (17.0%) participants, in whom a majority 41/51 (80.3%) were less than 5 years old. More than half 35/51 (68.6%) were febrile, 25/51 (49.0%) had impaired consciousness, and 6/51(11.8%) had respiratory distress. LDH was elevated in 17/51 (33.3%) of the participants, 47/51 (92.2%) had hyperlactataemia and 2/51(3.9%) hypoglycaemia.

Impaired consciousness was found in 50/300 (16.7%) participants. More than half—34/50 (68.0%) had reported vomiting, with 25/50 (50.0%) having had convulsions. A number of these patients had the following laboratory features: leukocytosis 26/50 (52%), hyperlactatemia 42/50(84%), hyperglycaemia 41/50 (82%), hypoglycaemia 5/50 (10%), and hyperpyrexia was in 6/50 (12.0%). Acidosis (lactic acidosis) was noted in 41/300 (13.7%) participants; the majority, 36/41 (87.8%), had severe pallor and 33/41(80.5%) jaundice. In addition, leukocytosis was observed in 26/41 (63.0%) and 06/41 (14.6%) had elevated lactic dehydrogenase (LDH).

Hyperparasitaemia was observed in 26/300 (8.7%) participants; 19/26 (73.1%) were below 5 years of age. All participants 26/26 (100%) presented with fever.

Respiratory distress was a finding in 20/300 (6.7%) participants, 8/20 (40%) of these had hyperlactatemia, 17/20 (85.0%) had impaired consciousness, and hypoxaemia was observed in 3/20 (15%). Only one patient had pneumonia as a co-infection.

Cerebral malaria was recorded in 18/300 (6.0%) participants and, out of these, only one (1/18) was hypoglycaemic. However, for technical reasons, we did not carry out lumber punctures to rule out any other causes of coma.

Impaired renal function was observed in 15/300 (5.0%) participants. Six out of 15 (40%) were reported to have passed dark urine. Jaundice, severe pallor and profound anaemia were present in all 15 participants. All had elevated BUN > 20 mg/dL and raised creatinine levels on laboratory evaluation. One progressed to acute kidney injury with anuria and electrolyte derangement. However, all recovered without progression to full-blown renal failure. Hypoglycaemia (random blood sugar < 2.2 mmol/L) was an uncommon feature in this study, observed in only 14/300 (4.7%).

Spontaneous bleeding was in only 9/300 (3.0%) participants who had reported spontaneous bleeding. All these participants had normal lactate and blood sugar levels, and none had thrombocytopenia. Only one out of nine had raised creatinine, elevated liver enzymes and the presence of respiratory distress. The bleeding episodes included epistaxis (2/9), haematemesis in 2/9 participants, and 5/9 had melaena. None had prolonged bleeding from injection sites.

The hypovolemic shock was in only two 2/300(0.7%) participants. This was the least common of all features. Both had jaundice with severe conjunctival pallor, temperature gradient, tachycardia and capillary refill time > 3 s. Laboratory parameters indicated severe malaria anaemia, leukocytosis, and normal platelet count with elevated LDH and bilirubin levels.

**Fig. 2:**
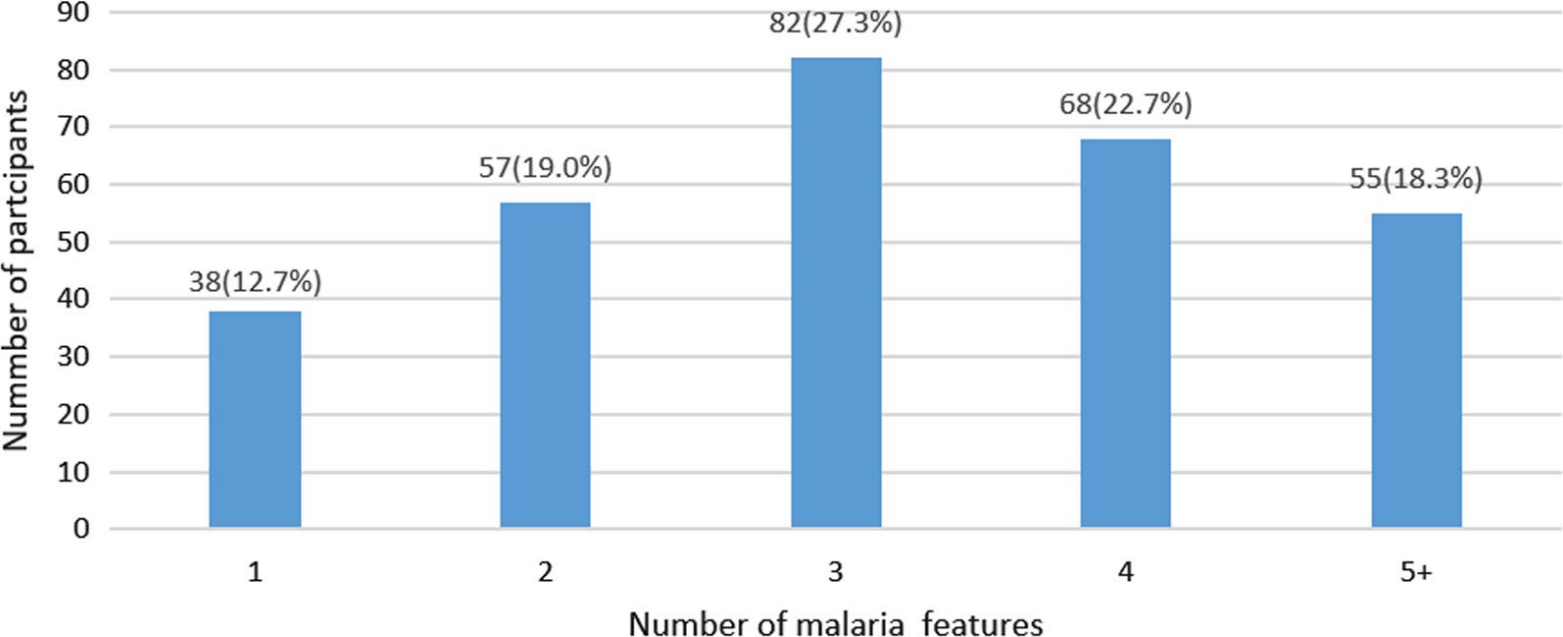
Combination of severe malaria features

Some of the participants had multiple severe malaria features. Figure 2 shows that several patients presented with more than one severe malaria feature. Of these, 57/300 (19.0%) presented with two features, 82/300 (27.3%) with three features, 68/300 (22.7%) with four features and 55/300 (18.3%) with five or more features.

In addition, the mean of the haematological, biochemical and vital results were as follows; haemoglobin 5.8 g/dL, white blood cells(total) 10.2 × 10^3^/ul, glucose 5.0 mmol/L, blood lactate 2.8 mmol/L, BUN( blood urea nitrogen) 16.75 mg/dL, temperature 37.8 ℃, and Blantyre coma scale had a median of 5.

### Outcomes

Out of 300, 251 (83.7%) stayed up to discharge, and these were analysed for prolonged hospitalization, 34 (11.3%) were self-discharges (runaways) and in-hospital mortality was14 (4.6%) and 28 day mortality was 19 (6.3%). Table 1 shows the association between severe malaria phenotypes and prolonged hospitalization. Impaired consciousness, lactic acidosis and respiratory distress were commonly associated with prolonged hospitalization. Table 2 is a multivariate table for mortality and shows that impaired consciousness (*P* = 0.016) and acidosis (*P* = 0.016) were independently associated with mortality.

**Table 1:** Association of severe malaria phenotypes with prolonged hospitalization

Variable	Overall n = 251	Hospital stay		OR (95% CI)	*P-* value
		< 5 days n = 195	> = 5 days n = 56		
Cerebral malaria	12 (4.8)	7 (3.6)	5 (8.9)	2.6 (0.8,8.6)	0.110
Multiple convulsions	41 (16.3)	32 (16.4)	9 (16.1)	1.0 (0.4,2.1)	0.952
Haemoglobinuria (dark urine)	130 (51.8)	97 (49.7)	33 (58.9)	1.4 (0.8,2.6)	0.227
Hypovolemic shock	2 (0.8)	1 (0.5)	1 (1.8)	3.5 (0.2,57.3)	0.376
Severe malaria anaemia	129 (51.4)	99 (50.8)	30 (53.6)	1.1 (0.6,2.0)	0.712
Prostration	195 (77.7)	147 (75.4)	48 (85.7)	2.0 (0.9,4.4)	0.106
Impaired consciousness	32 (12.8)	21 (10.77)	11 (19.6)	2.0 (0.9,4.5)	0.084
Spontaneous bleeding	8 (3.2)	5 (2.6)	3 (5.4)	2.1 (0.4,9.3)	0.305
Hypoglycaemia	7 (2.7)	5 (2.6)	2 (3.6)	1.4 (0.3,7.5)	0.688
Respiratory distress	11 (4.4)	6 (3.1)	5 (8.9)	3.1 (0.9,10.5)	0.072
Acidosis (lactic acidosis)	26 (10.4)	16 (8.2)	10 (17.9)	2.4 (1.0,5.7)	0.041
Renal failure	9 (3.6)	5 (2.6)	4 (7.1)	2.9 (0.8,11.2)	0.119
Hyperparasitaemia	22 (8.8)	19 (9.7)	3 (5.4)	0.5 (0.1,1.8)	0.314
Jaundice	170 (67.7)	128 (65.6)	42 (75.0)	1.6 (0.8,3.1)	0.189

**Table 2:** Multivariate table for mortality

Variable	COR (95% CI)	*P*-value	AOR(95% CI)	*P*-value
Cerebral malaria	5.4 (1.7, 17.3)	0.005	1.5 (0.4, 6.4)	0.556
Impaired consciousness	8.3 (3.2, 21.4)	0.001	4.8 (1.3, 17.0)	**0.016**
Respiratory distress	6.5 (2.2, 19.7)	0.001	1.1 (0.3, 4.4)	0.941
Acidosis (lactic acidosis)	6.9 (2.7, 18.0)	0.001	3.9 (1.3, 11.9)	**0.016**

## Discussion

Malaria incidence (i.e. cases per 1000 population at risk) reduced from 80 in 2000 to 58 in 2015 and 57 in 2019 globally [1, 8]. Despite these gains, the malaria situation in sub-Saharan Africa (SSA) remains volatile [9]. During the COVID-19 pandemic, there was a reversal of earlier reported gains in a reduction in mortality, with an excess of 69,000 deaths reported in the SSA in 2020 [2, 10]. This vulnerability seems to be driven by multiple factors that have shaped the dynamics of the malaria epidemiological quartet of vector, agent, host and environment [11]. The emergence of novel malaria-transmitting mosquito *Anopheles stephensi* mosquito in SSA [12], has posed a new threat to malaria control measures. In addition, the recent emergence of independent malaria drug resistance in Rwanda [13], Tanzania [14], and Uganda [15] has caused challenges in malaria control using artemisinin-based combination therapy (ACT).

There are also changing characteristics of the host. In recent studies, whereas children remain vulnerable to malaria, the age strata and clinical disease picture have changed [16–18]. Furthermore, tropical weather patterns and topography of marshlands accentuated by global warmings seem to improve the breeding of *Anopheles* mosquito and enhanced efficiency for its disease transmission capabilities in the last decade [19–21]. A constellation of these factors seems to have ushered in a new era of malaria in SSA, associated with variations in the clinical spectrum of severe malaria, but this has remained hitherto poorly described.

A total of 300 children, randomly selected among children being recruited into a longitudinal malaria study. This is a much larger sample size compared to recent similar studies for the work we report here [22]. Therefore, these new phenomena represent a more widespread change in malaria presentation during the malaria epidemic in the paediatric population of Eastern Uganda.

In this study, the common clinical features reported were prostration 236/300 (78.7%), jaundice 205/300(68.3%), severe malarial anaemia 158/300(52.7%), black water fever (BWF) 158/300 (52.7%), and multiple convulsions 51/300 (17.0%). Prolonged hospitalization was found in 56/251(22.3%). Participants with acidosis were more likely to have prolonged hospitalization *P* = 0.041. More than half 154/300 (51%) of the study participants had already been treated with either oral or parental anti-malarial. Our data show that the proportion of patients with each clinical feature has changed with some increasing tremendously compared to similar data reported in the same setting a decade ago [5]. It is plausible that there was an exponential increase in cases of malaria during the malaria epidemic that the Uganda Ministry of Health declared in June 2022. However, this does not necessarily explain the change in the clinical spectrum from the predominant respiratory distress and severe malarial anaemia a decade ago [19] to a higher proportion of children with prostration and BWF in the current data. This demonstrates a shift in age strata towards older children. Elsewhere, malaria presentation in older children is commonly associated with more cerebral and renal involvement compared to frequencies of these features in younger children [23]. Previous data highlighted that BWF is increasing in Eastern Uganda [18], but this time round, this report shows that it has become even more common. In the earlier and current data, there is an increase in the average age of children suffering from severe malaria. Even though BWF seems to be increasing, especially in Eastern Uganda [18], the pathophysiology is typically different and depends on clinical presentation. Our data demonstrate a triad of severe anaemia, jaundice and BWF, which point towards massive haemolysis.

Conversely, BWF associated with cerebral malaria [23, 24] is a pointer towards myolysis, as myoglobin has previously been detected in children with impaired consciousness and BWF [24]. The pathogenesis of cerebral malaria is due to damaged vascular endothelium by parasite sequestration, inflammatory cytokine production and vascular leakage, which result in brain hypoxia, as indicated by increased lactate and alanine concentrations [25]. It is possible the source of myoglobin in the children with cerebral malaria is multifactorial. Firstly, the muscle damage from generalized tonic–clonic seizures. Secondly, vaso-occlusive phenomenon occurs in muscle blood supply following damaged vascular endothelium by parasite sequestration similar to cerebral malaria. In Eastern Uganda, previous data consistently reported a low frequency of impaired consciousness in severe childhood malaria even with changes in age strata [5, 18, 26].

Other clinical observations in our current study may suggest that earlier treatment with antimalarials may be responsible for the variation in the clinical spectrum and outcomes observed. Over one-half of the study participants had taken anti-malarial before admission. In SSA, there is a paucity of data on the role of ACT in the causation or prevention of BWF. Earlier data reported that BWF was associated with quinine, the first-line drug for treating severe malaria two decades ago [26] or where it is still in use [27]. There have been postulations that artemisinin-based combinations fast-acting intra-erythrocyte activity results in death to malaria parasites, causing pitting of the infected red blood cells and resulting in premature apoptosis, autoimmune and spleen-driven lysis, culminating in massive haemolysis [28]. In some settings, case studies have indicated that ACT could be associated with BWF [29], but further research is needed in these setting. The presence of a rising proportion of BWF could as well be explained by presence of red cell abnormalities probably undiagnosed in this community. Olupot-Olupot et al. [18] had earlier reported prevalences among his patients for HbAS 4.6%, HbSS 4.1%, alpla-thalassaemia homozygous in 3.7% and heterozygous 38.0% and G6PD deficiency in 15.6%.

In this study, the median age was 4.6 years and a proportion of children below 5 years of 164/300 (54.7%). This demonstrates a shift towards older children and a lower proportion of children under the age of 5 years compared to a similar study in the same settings by Olupot-Olupot et al*.* [5] that reported an average age of 1.5 years for data collected a decade ago. Elsewhere, Kalinga et al. [30], assessed clinical manifestations and outcomes of severe malaria in children admitted to district hospital in Rungwe and Kyela in south-western Tanzania [30]. In their study, 1371 children were selected as screening group of which 409 (29.8%) tested positive for malaria. The mean age of the children was 2.7 (95%CI = 2.5, 2.8) years, and the majority (86%) were under 5 years of age [30]. These results may propose that the current epidemic of malaria-affected older children with waned immunity compared to earlier studies in which younger nonimmune children were affected. It is plausible that these age differences are also responsible for the differences in the clinical spectra of the disease between earlier and current malaria series.

In most malaria series, low rates of clinical jaundice have been reported. In Eastern Uganda, for instance, in 2012 Olupot-Olupot and colleagues [5] reported that jaundice accounted for 26.7% of children with severe malaria and now this report demonstrates a higher proportion 205/300 (68.3%) in the same settings. Therefore, this suggests that massive haemolysis is the underlying cause given the unique triad of severe anaemia, jaundice and BWF reported in the earlier studies [18] and the current data.

In Eastern Uganda, cerebral malaria has consistently remained low [18]. In the current data, cerebral malaria was in 10/137(6.0%) despite the older age of children with severe malaria in this study population. On the other features, the current data reports a reversal in the rates for respiratory distress. For instance, respiratory distress of 6.7% was much lower than in earlier series in the same settings [5, 18, 26]. The main difference is age, and it is well documented that respiratory distress is common in severe malaria in younger children [5]. The transition from quinine to artesunate could have influenced the change in the spectrum especially since artesunate has a rapid parasite clearance and the fact that more than half of the participants had pre-referral artemisinins. This could have reduced many incidences of cerebral malaria especially in the older children. There is also a possibility of increased haemolysis of the pitted red blood cells, which could be contributing to the rising jaundice and BWF with associated severe anaemia in these children. However, there is a paucity of data supporting this as earlier mentioned and more research is warranted especially in the most affected communities in Eastern Uganda.

On the outcomes, 52/251 (22.3%) study participants had prolonged hospitalization, similar to other series previously reported, participants with acidosis (*P* = 0.041), were more likely to have prolonged hospitalization. The overall in hospital mortality was 4.6%, similar to the 4.6% reported in Tanzania [30] but lower than earlier reports by Olupot-Olupot et al*.* in the same settings [5, 18, 26].

A retrospective study on adherence to malaria case management at this study site conducted between June 2021 and March 2022 indicated poor adherence to malaria treatment resulted in higher morality at 40/147 (27%) [22]. The difference with the current data could be attributed to several factors and the review period corresponded to the tail end of the COVID-19 lockdown. During this period, it was observed that few, but mainly very critically sick children accessed referral services at this facility, few health workers were working, and drug supplies were erratic as an effect of COVID-19. This effect of COVID-19 on malaria outcomes has been reported previously [2]. Compared to pre-COVID-19 of 63/662 (9.5%) for a similar study done May, 5 2011 until April,30 2012 in Eastern Uganda [5], the mortality in hospital mortality was much lower. This mortality in the first post-COVID-19 lockdown year is an indication of improvement attributable to recovery in the case management process. In addition, the older average age of the study participants in the current data and high rates (> 50%) of pre-admission anti-malarial treatment may have contributed to the observed outcomes.

Overall, the factors that were independently associated with mortality in this study were acidosis (*P* = 0.016) and impaired consciousness (*P* = 0.016). These same factors have been reportedly associated with poor outcomes in African children with severe malaria [31–33]. In addition, there were many runaways (self-discharges) whose outcomes could not be ascertained in their community. Against this background, the data on outcomes is incomplete. However, this has demonstrated that in the post-COVID-19 malaria epidemic, the clinical spectrum of disease is unique and mortality outcomes remain poor.

The limitations were; short duration, small sample size, lumbar puncture/CSF studies and blood cultures were not done. Adherence to anti-malarial therapy could not be closely tracked and neurological sequelae of severe malaria were not looked for and documented.

## Conclusion

These findings indicate a changing trend in the clinical spectrum of severe malaria associated with the disease epidemic and older age. There were more children with prostration, BWF and jaundice. Pre-referral treatment may play a role in both the clinical spectrum of disease and outcomes. Further research is needed in other populations to see if these findings are more widespread.

## Data Availability

The study data are available by request to the corresponding author.
